# High-Throughput Rapid and Inexpensive Assay for Quantitative Determination of Low Cell-Density Yeast Cultures

**DOI:** 10.3390/microorganisms7020032

**Published:** 2019-01-24

**Authors:** Debora Casagrande Pierantoni, Laura Corte, Luca Roscini, Gianluigi Cardinali

**Affiliations:** 1 Department of Pharmaceutical Sciences, University of Perugia, 06123 Perugia, Italy; debora.casagrandepierantoni@gmail.com (D.C.P.); Laura.corte@unipg.it (L.C.); 2 CEMIN, Centre of Excellence on Nanostructured Innovative Materials, Department of Chemistry, Biology and Biotechnology, University of Perugia, 06123 Perugia, Italy; luca.roscini@unipg.it

**Keywords:** *Candida* spp., cell density, densitometry, high-throughput, lag phase, yeast

## Abstract

A procedure for microbial cell density determination with a high-throughput densitometric assay was developed to allow a precise quantification of both free and sessile cells, such as those of a biofilm, with a large range from low to high cell densities. Densitometry was chosen because it allows fast, rapid and cost-effective measures; it is non-disruptive; and has an easy learning curve. The method setup, and the further validation, was carried out with strains of *Candida albicans, C. tropicalis* and *C. parapsilosis.* Equations were developed at the level of the single strains, of the three species and finally a general one applicable to all three species. In the cross validation, with strains absent from the training set, the method was shown to be robust and flexible. The best results were obtained with species specific equations, although the global equation performed almost as well in terms of correlation between real and estimated density values. In all cases, a correlation around 0.98 between effective and predicted density was obtained with figures ranging from 10^2^ to 10^8^ cells mL^−1^. The entire analytical part of the procedure can be accomplished with a MS Excel macro provided free of charge.

## 1. Introduction

The determination of the cell number is one of the most venerable microbial techniques dating back to early times of microbiology. Microbiologists have since developed many techniques and variants to count the viable as well as the total cells onto surfaces and in liquids. Viable cell counts is the oldest approach and shows a detection limit as low as ca. 30 cells mL^−1^, but is affected by several drawbacks such as the impossibility to count Viable Non-Culturable (VNC) strains, the need of specific media, the time necessary for the cell growth, the labor intensity and the low susceptibility to high throughput [1,2,3,4]. On the contrary, indirect total counts are rapid, insensitive to VNC and amenable to high throughput settings [2,5], but suffer in terms of detection and of density range [6]. Some of these techniques are complex and expensive in terms of consumables and dedicated equipment such as Real Time PCR (RT-PCR) [7,8,9,10] or some fluorescent based techniques [11]. Others are relatively inexpensive, such as the turbidometry in multi-well plates, but suffer from a relatively high detection limit and have problems when cell density covers several orders of magnitude, as normally happens when there is the need for following a growth curve from very low to very high cell densities [12,13]. In fact, high cell density values can be detected spectrophotometrically but the linearity of the method decreases with the increase of the cell densities [14]. One can overcome the problem by serial diluting the cell suspension, but this would make the procedure even more complex and laborious.

When cell counting is applied to cells on surfaces, such as microbial biofilms [15,16], the problems are made more complex by the necessity of detaching the cells from the surfaces, implying that some counting could be affected by underestimation, due to the low efficacy of the detaching treatment [17]. These methods can be applied for research in a microbiology laboratory, but most would be inappropriate for applicative environments, where high throughput and a swift procedure are as essential as low cost per operation [18,19,20]. This can be the case of monitoring [21] for the presence of cells, even at low densities, in biotechnological industries.

Based on these observations, the present study aimed at developing a high throughput turbidometry-based method able to determine microbial cell densities with a large range of several orders of magnitude, amenable to being used in research and industrial environment for the detection of both planktonic and sessile cells. In this frame, the novelty of the approach is to use the growth curve kinetics to determine low-density inocula, whereas relatively high-density inocula can be determined with a simple turbidometry based calibration curve. The relatively low cost and handiness of multi-well plates for the densitometric reading at 405 nm allows their use in many applicative settings as well as in research laboratories. Moreover, this type of plate is the gold standard for the study of biofilm development in both bacteria [22] and fungi [23], making it the ideal tool adaptable to both planktonic and sessile cell counting. The rationale of this paper is that the growth curve derived from the cells present in each single well of a plate will have kinetic characteristics useful to determining the actual densities of those cells.

## 2. Materials and Methods

### 2.1. Materials

#### 2.1.1. Strains

The nine strains employed in this study (Table 1) were obtained from the CEMIN Microbial Collection (CMC, CEMIN Excellence Research Centre—University of Perugia). All strains were isolated from nosocomial environments (Table 1) and afterwards kept frozen at −80 °C in 17% glycerol [24]. All strains were identified by MALDI-TOF MS (Matrix Assisted Laser Desorption Ionization–Time Of Flight Mass Spectrometry) and sequencing of the D1/D2 domain of 26S rDNA subunit (LSU) and Internal Transcribed Spacer (ITS) markers [25]. The strains included three *Candida albicans* (CMC1959, CMC1968 and CMC2046), three *Candida parapsilosis* (CMC1949, CMC1972 and CMC1981) and three *Candida tropicalis* (CMC1827, CMC1839 and CMC2052) strains.

#### 2.1.2. Growth Media

All components of YEPD medium (Yeast Extract 1%, Peptone 1%, and dextrose 1%) were purchased from Biolife (Milan, IT) and RPMI-1640 was obtained from Sigma Aldrich (Milan, IT).

#### 2.1.3. Instruments

Tecan Infinite F-200 was acquired from Tecan Group (Tecan Trading AG, Männedorf, Switzerland).

### 2.2. Experimental Part

#### 2.2.1. Experimental Design

The rationale of this study is that the growth kinetic reveals useful metrics for estimating the inoculum size, even if the cells are attached to a solid surface, as in the case of sessile biofilm cells or cells of a thin pellet obtained by simple centrifugation or natural decantation.

#### 2.2.2. Experimental Procedure

Pre-cultures were obtained by inoculating a loopful of each sample into 7 mL YEPD medium and grown 24 h at 37 °C (120 rpm). Biomass concentration was determined using a Thoma-Zeiss counting chamber and then diluted in YEPD and RPMI-1640 media to the final concentrations from 5 × 10^7^ to 1.19 × 10^1^ cells mL^−1^. All dilutions were obtained directly in 96-well plates in a final volume of 100 µL.

CMC2046, CMC1949 and CMC1839 (*C. albicans, C. parapsilosis* and *C. tropicalis*, respectively) strains were used to validate the protocol at four different cell densities (6.25 × 10^6^, 1.95 × 10^5^, 1.22 × 10^4^ and 1.53 × 10^3^ cells mL^−1^, respectively). The 96-well plates were incubated in standardized conditions (24 h, 37 °C) using the multi-well plate reader Tecan infinite F-200. OD_405nm_ values were collected every 5 min, using the internal software of the instrument (Magellan^TM^, Tecan Trading AG, Männedorf, Switzerland). All analyses were conducted in triplicate.

### 2.3. Apparent Lag Phase Extent Definition

The Apparent Lag Phase Extent (ALPE) determination is based on the definition of lag phase itself. For this purpose, three different definitions of lag phase were considered for the development of the three algorithms used to calculate the lag phase length.

**Figure 1:**
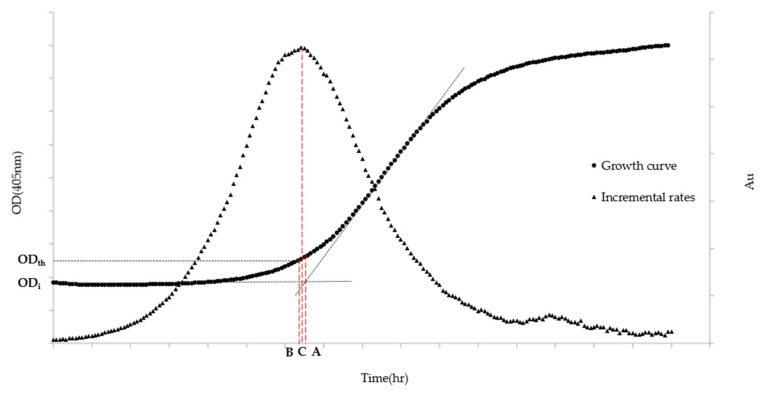
ALPE representation based on Algorithms A–C. OD_th_ stands for the Optical Density chosen by the analyst to be the threshold value, while OD_i_ indicates the Optical Density at the inoculum time. (A) ALPE using Algorithm A; (B) ALPE using Algorithm B; and (C) ALPE using Algorithm C. The left axis refers to the growth curve data (Optical Density) while the right one (Arbitrary Units) to the Incremental rates.

Algorithm A is based on the most widespread definition that describes the lag phase as the transition period during which the specific growth rate increases to the maximum value characteristic of the culture environment [26]. The ALPE is then calculated, on the typical sigmoidal-like growth curve, as the time equivalent at the intersection of the regression curve of the lag phase and that of the exponential phase. Algorithm A (Figure 1) is explained in the following equations:

(1) $$y_{1}{=\mathrm{OD}}_{i}$$

(2) $$y_{2}=\alpha \times t+\beta$$

The intercept of the two curves can be calculated as

(3) $$y_{1}{=y}_{2}$$

(4) $${\mathrm{OD}}_{i}=\alpha \times t+\beta$$

(5) $$t=\frac{{\mathrm{OD}}_{i}-\beta }{\alpha }$$

Algorithm B (Figure 1) is built on the definition that identifies the end of the lag phase as the time at which the initial optical density exceeds a predetermined growth.

This algorithm is based on the following equation:

(6) $$\mathrm{ALPE}\;=\;{\mathrm{OD}}_{i}\;\times \;\mathrm{Th}$$

where Th is the value chosen by the analyst.

The definition chosen to build Algorithm C (Figure 1) identifies the end of the lag phase as the maximum point of the “incremental rate” function calculated with the OD_405nm_ values.

(7) $$f^{\prime }=\frac{{\mathrm{OD}}_{\left(k+n\right)}-{\mathrm{OD}}_{\left(k\right)}}{t_{\left(k+n\right)}-t_{\left(k\right)}}$$

where k indicates the kth OD_405_ value. The algorithm calculates the difference in OD of two values 10 sampling points apart and produces the function with a moving window mechanism of size n, normally set as n = 10. Increasing the n value tends to smooth the curve, whereas lower values introduce some additional noise.

(8) $$\mathrm{Max}\left(f^{\prime }\right)=\mathrm{ALPE}$$

### 2.4. Initial Cell Density (IND) Calculation

IND is calculated taking alternatively into account both ALPE and the initial OD_405_ in the following pair of equations

(9) $$\mathrm{IND}=10^{\left(\alpha \;\mathrm{ALPE}i+\beta \right)};\;\mathrm{if}\;\mathrm{ALPE}\;>\;k$$

(10) $$\mathrm{IND}={\gamma \;\mathrm{OD}}_{405}+\delta \;\mathrm{IND};\;\mathrm{if}\;\mathrm{ALPE}\;\leq \;k$$

where *α* and *β* represent the angular coefficient and the intercept of the calibration curve obtained in the regression curve of actual cell densities vs. ALPE. Similarly, *γ* and *δ* are, respectively, the angular coefficient and the intercept of the regression curve comparing actual cell densities and OD_405_ values.

Equation (10) is used when the initial optical density (OD_405_) exceeds the reader’s lower detection limit. The k value was empirically set at 30 and 100 min for Algorithms A and B, respectively. For Algorithm C, there is no need of this offset value and k = 0.

### 2.5. Statistical Tools

Analyses were carried out in Microsoft Excel, using the native editor for VBA programming. Regression curves were created and analyzed with the native Excel functions SLOPE and INTERCEPT.

## 3. Results

### 3.1. The Optical Density Is Not Indicative of the Initial Cell Content

When trying to estimate the total cell density, the most obvious rationale is to calculate it with a regression curve based on the optical density of the cell suspension. This is possible as long as a linear, or at least a monotonic relationship, exists between optical density and total cell density.

**Figure 2:**
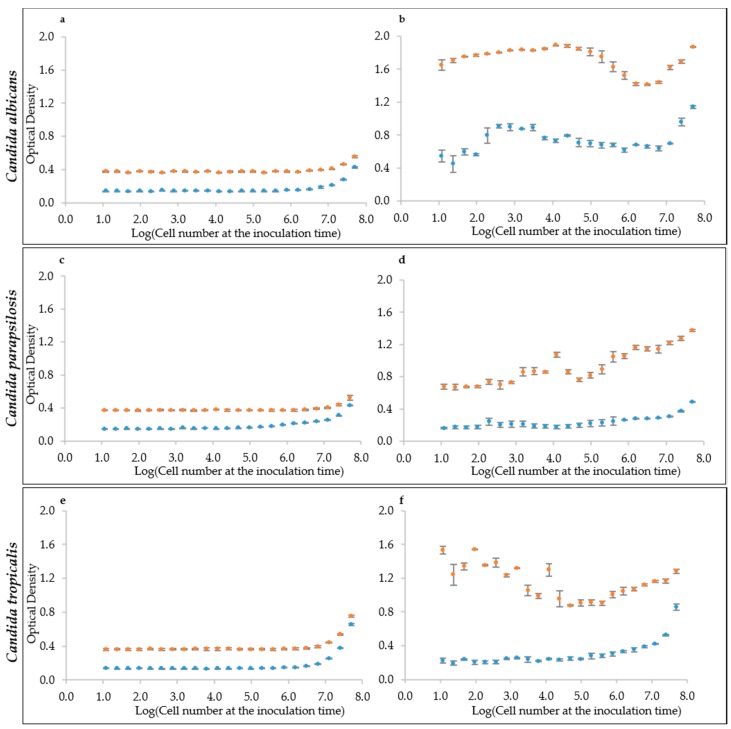
Optical Densities distribution at the inoculum time and after 24 h growth. (**a**,**c**,**e**) Inoculation time cell densities for the species: (**a**) *C. albicans* (CMC1698); (**c**) *C. parapsilosis* (CMC1981); and (**e**) *C. tropicalis* (CMC1827). (**b**,**d**,**f**) Optical density values after 24 h growth. RPMI medium is represented by the light-blue marker while YEPD by the orange one.

We tried this approach to evaluate the initial cell density in multi-well plates, which is the situation of an initial biofilm adhesion or of any cell culture starting at very low density. To assess the linearity between optical and cell density, we tested a series of samples at increasing cell density spanning from ca. 10 to 10^8^ cells mL^−1^. The resulting optical density (OD_405_) did not increase until cell densities reached the order of 10^6^ in both YEPD and RPMI medium, for all six strains of the three species *C. albicans* (Figure 2a and Figure S1a), *C. parapsilosis* (Figure 2c and Figure S1c) and *C. tropicalis* (Figure 2e and Figure S1e). This finding ruled out the hypothesis of using the raw optical density, as obtained by the multi-well plate reader, to estimate the initial cell density of a sessile or of a planktonic culture. The density of 1 million cells mL^−1^ represents the detection limit for yeast cells of most of the common plate reader using these specific settings. An alternative hypothesis is to assume that a standardized growth could act as a sort of amplifier of the initial cell density of the culture. For this reason, the optical density of the same cell suspensions in YEPD and RMPI medium was read after 24 h of growth. Results indicate that indeed there was an amplification effect, but not always with the same response by the various strains in the two different media. Namely, *C. albicans* in YEPD displayed a non-monotonic trend with a peak of the cultures starting around 10^4^ cell mL^−1^ (Figure 2b and Figure S1b). A similarly non-monotonic behavior was shown by *C. tropicalis* (Figure 2f and Figure S1f). On the contrary, *C. parapsilosis* had an almost monotonic trend in YEPD, with some exceptions (Figure 2d and Figure S1d). In general *C. parapsilosis* and *C. tropicalis* cells grew poorly in RPMI, thus producing a very little increment (when present), in comparison with the reading at the beginning of the culture. Interestingly, RPMI was more effective at enhancing *C. albicans* growth, but did not produce a monotonic trend (Figure 2b and Figure S1b).

### 3.2. The g Time Is Not Indicative of the Initial Cell Content

Based on the above-mentioned consideration that the growth of a culture has kinetic factor allowing to estimate the initial cell number, or density, the g time was calculated. Although the generation time should be rather constant throughout the exponential phase, the rationale of this experiment is that higher cell numbers should guarantee a more rapid entrance into the active growth and therefore induce a higher g time. The experiments, carried out with the same settings of the previous section, showed that the g time underwent some variations, but it was clearly independent from the initial inoculation density. Only the *C. parapsilosis* strains in YEPD showed hints of linearity in the range from 10^5^ to 10^8^ cell mL^−1^ and *C. tropicalis* between 10^6^ and 10^8^ in the same medium. In all other conditions, the g time varied from 0.5 to 1 h, regardless of the inoculation density (Figure S2).

### 3.3. The Apparent Lag Time Is A Predictor of the Inoculum

**Figure 3:**
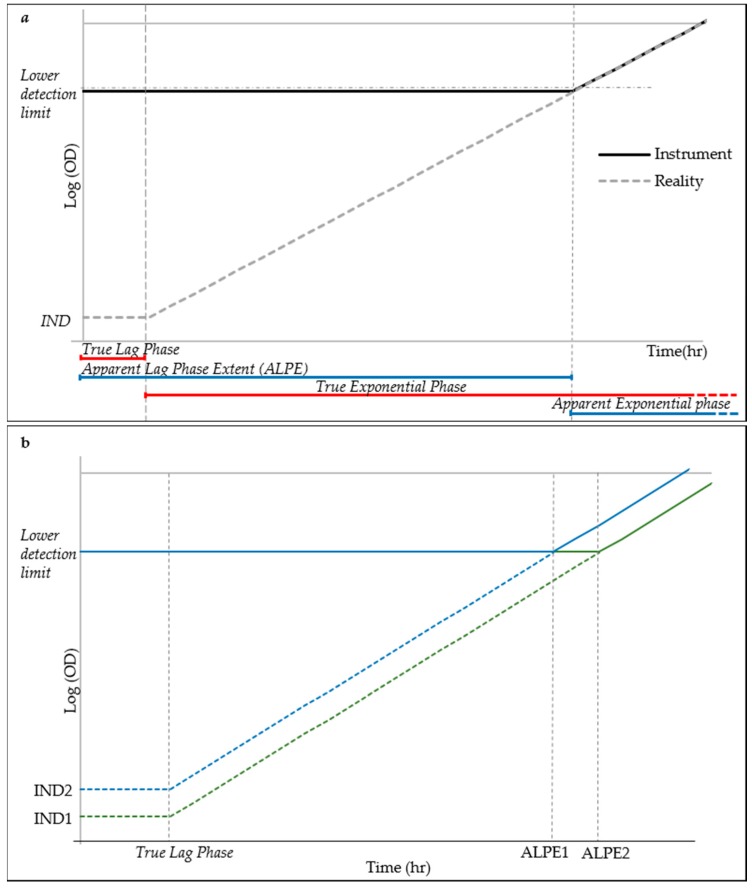
Simplified model of cell growth for ALPE explanation: (**a**) schematic explanation of the Apparent Lag Phase Extent (ALPE), where IND indicates initial cell density; and (**b**) simplified growth curves (in green and blue markers) from two different inocula concentrations corresponding to different ALPE.

It is well known that the lag phase precedes the exponential growth and it is characterized by a stability of the live cell density and therefore also of the total cell counting. The motivation of using the lag phase duration as a predictor of the initial cell density of the inoculum is explained in the simplified model of cell growth displayed in Figure 3. Low inoculum density is below the resolution of the optical density reader, as demonstrated by the experiments in Figure 2. This situation causes the early exponential phase to be detected as a continuation of the lag phase, until the cell growth has not reached the lower detection limit of the detector when the exponential phase appears, much after its real start. The lag phase, as detected by the reader, hereinafter referred to as “apparent lag phase”, is therefore the real lag phase and part of the early exponential phase (Figure 3a). Under this model, assuming a constant lag phase and **g** time, the time necessary for a culture to reach the lower detection limit would therefore be dependent on the initial density (IND) (Figure 3b). Lower inoculation densities will cause longer apparent lag phase extent (ALPE), whereas higher inocula close to or above the lower detection limit of the reader will induce short lag phases.

**Figure 4:**
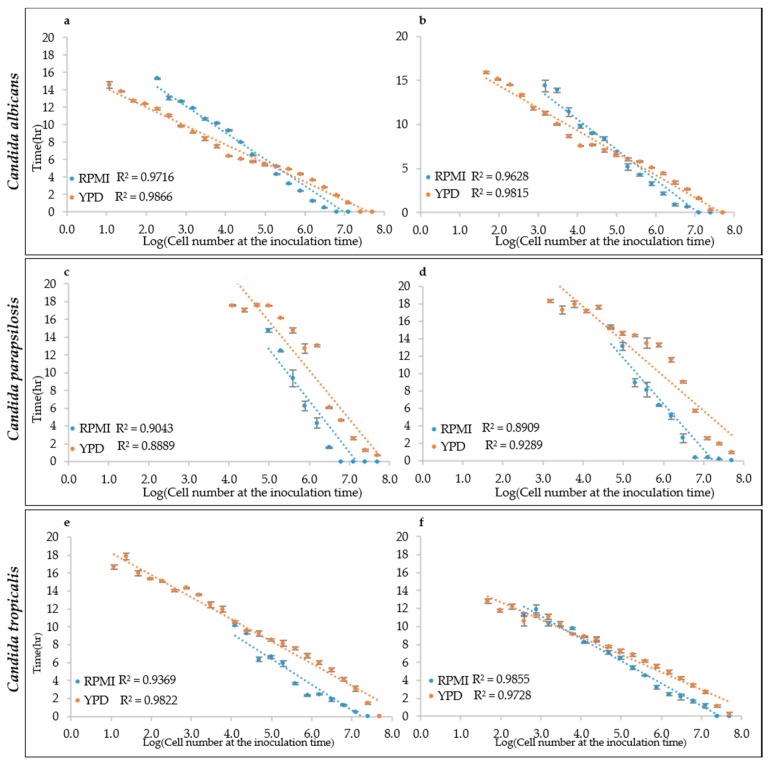
ALPE determination: (**a**,**b**) *C. albicans* CMC1968 and CMC1959, respectively; (**c**,**d**) *C. parapsilosis* CMC1972 and CMC1981, respectively; and (**e**,**f**) *C*. *tropicalis* CMC 1827 and CMC2052, respectively.

To test this hypothesis, the growth curves obtained from different inoculation cell densities were used to determine the ALPE values by visual inspection. Results show that indeed the ALPE was negatively well correlated with the inoculum densities (Figure 4). In *C. albicans* strains, the R^2^ of the regression curves ALPE vs. log of the inoculum densities were higher with YEPD than with RPMI (0.9866 and 0.9815 vs. 0.9716 and 0.9628). The linear relationship covered the whole span from 10 to 10^7^ in RPMI and 10^8^ in YEPD (Figure 4a,b). The good relationship between ALPE and IND was present in the two other species, although with some variation. In *C. parapsilosis*, the R^2^ values were lower than in *C. albicans* and YEPD was not always better than RPMI (Figure 4c,d). Conversely, *C. tropicalis* showed a situation similar to *C. albicans*, although the better performance of YEPD was confirmed only in the strain CMC 1827 (Figure 4e,f).

### 3.4. Three Different Approaches to Calculate the ALPE

**Figure 5:**
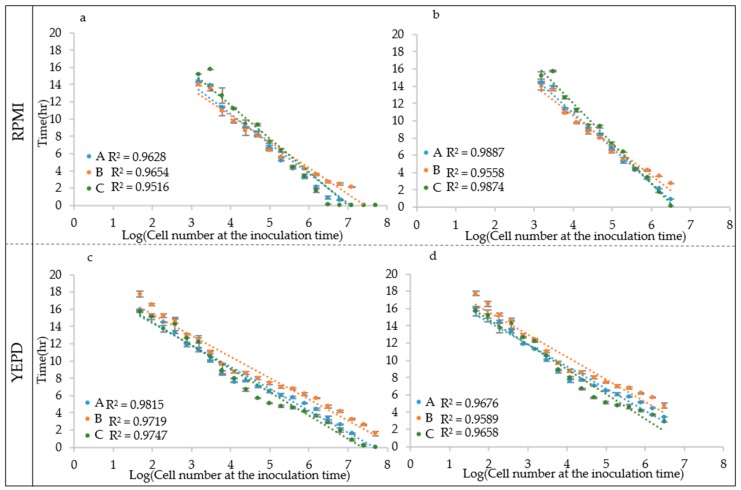
*C. albicans* CMC1959 ALPE. ALPE was calculated using Algorithm A–C. Regression analysis was carried out using: the whole IND range (**a**,**c**); and the reduced range (**b**,**d**).

The accurate determination of the IND relies on a model considering the ALPE as well as the initial OD_405_ for those cases in which the Optical Density of the IND is above the minimum detection limit of the reader and the apparent lag phases is particularly short. For this purpose, a pair of equations (Equations (9) and (10)) was proposed to consider both factors and guarantee a good evaluation of the IND in all cases. In this framework, the determination of the ALPE was one of the most challenging aspects; in fact, the shape of the curve in the transition from the lag to the exponential phase does not display a blunt flex point and, in some cases, it turned out that the visual definition of the lag phase could be questionable. This consideration led to the development of three approaches to evaluate the ALPE. The first (Algorithm A) is based on the long-standing concept that the transition between the two phases can be determined by the intersection of the regression curve of the lag phase and that of the exponential phase (Figure 1). In a more simplistic way, Algorithm B defines the beginning of the exponential phase as the time when the optical density exceeds a predetermined threshold. This algorithm relies strongly on the experience and observation capability of the analyst but fits well to certain growth curves with a long transition from the apparent lag to the apparent exponential phase (Figure 1). Finally, Algorithm C calculates the incremental rates of the growth curve in a short range (few minutes, determined by the analyst) in a sliding window manner, producing a curve the maximum of which is a good determination of the end of the apparent lag phase (Figure 1). The use of the three algorithms produced three different series of ALPE estimates from the growth curves obtained with different IND values. In general, the regression curves showed a good fitting (R^2^ > 0.93) between IND and ALPE (Figure 5). The R^2^ values obtained with RPMI were very similar to those from YEPD (0.95 vs. 0.96). Similarly, the three algorithms showed comparable R^2^ values, i.e., 0.96, 0.96 and 0.95 for Algorithms A–C, respectively. A loss of linearity was detectable at the higher IND values (Figure 5a,c), suggesting that the ALPE could be a good descriptor to estimate medium or low levels of cell density. To test this hypothesis, the IND range was reduced (Figure 5b,d), with an increase of the R^2^ values from the RPMI test (Figure 5b), but not from the growths on YEPD that instead showed either the same R^2^ values or some decrease, as in the case depicted in Figure 5d. Altogether, these results show that ALPE was well and negatively related to the initial cell density and that can be used in its estimate.

### 3.5. Models to Estimate the Cell Density

**Figure 6:**
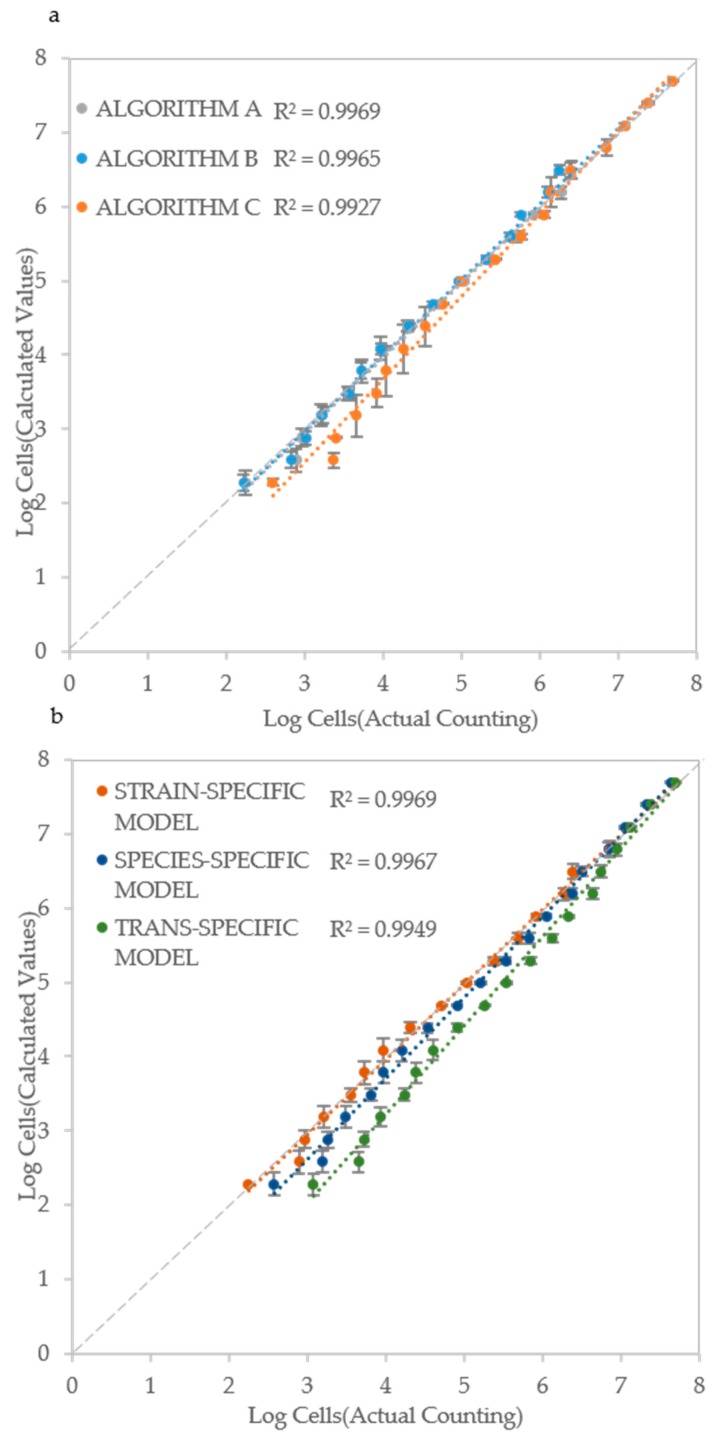

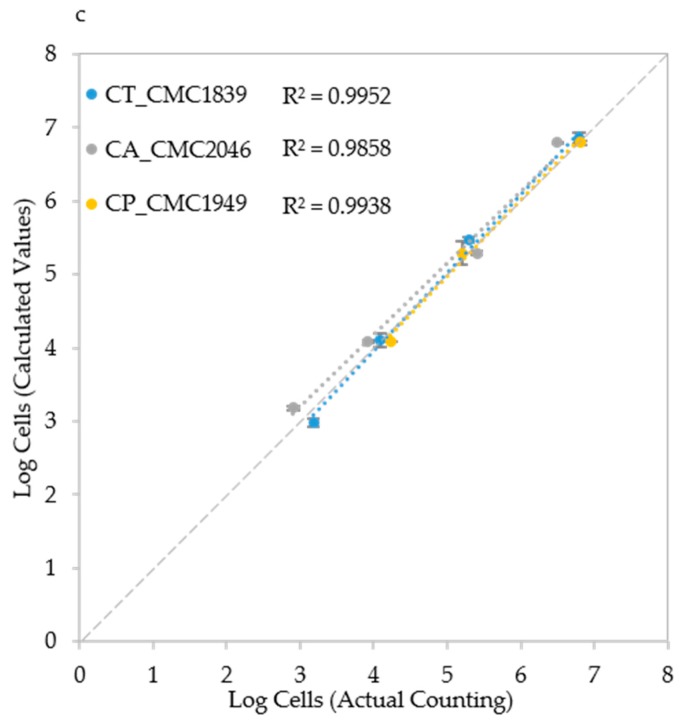
Cell number estimation and procedure validation: (**a**) Comparison of the three algorithms in *Candida albicans* CMC1968; (**b**) Strain-specific, species-specific and trans-specific models used to estimate IND; and (**c**) validation of the procedure using species-specific model in *C. albicans* (CMC2046), *C. parapsilosis* (CMC1949) and *C. tropicalis* (CMC1839) strains. Actual cell densities were determined using a Thoma-Zeiss counting chamber.

Based on the ALPE determination, and of its good regression with IND values, a model (equation) was developed that takes in account the initial optical density and the ALPE. Considering that the optical density is a good measure of the cell density when it exceeds the lower detection limit of the instrument (see Figure 3), the rationale of the model is that the input can be the initial optical density for higher IND values and the ALPE for the lower ones. To test the validity of this approach, a strain specific model was developed for each strain and then used to evaluate the initial cell density. The inputs consisted of the initial optical density and of the ALPE values, calculated separately with the three algorithms. A typical result is shown in Figure 6a, where all three algorithms showed good performances with R^2^ ranging from 0.9927 (Algorithm C) to 0.9969 (Algorithm A), suggesting once again that all three approaches to calculate the ALPE are applicable.

The set-up of the model requires to read the initial optical density and to evaluate the ALPE of a series of initial cell densities, normally deriving from a 1:2 dilution. Although the procedure is simple and rapid, the possibility to develop species-specific and general (trans-specific) models would allow for a swifter application of the proposed method. The species-specific model was obtained by averaging the strain-specific models of all strains of the same species, whereas the trans-specific model was obtained by averaging all the strain specific models. The concept of this extended approach is illustrated in Figure 6b, where the data of *C. albicans* CMC 1968 were introduced in its strain specific model, then in the *C. albicans* specific model and finally in the trans-specific model, obtaining regressions with R^2^ ranging from 0.9949 to 0.9969, using the sole Algorithm A.

In each of the strain-, species- and trans-specific models, the ALPE can be calculated with one of the three algorithms described above. The combination of algorithm and model was applied to all tested strains in both RPMI and YEPD as depicted, respectively, in Table 2 and Table 3.

The strain specific models performed well with R^2^ values as low as 0.9561 in RPMI (Algorithm B) and 0.8168 in YEPD (Algorithm C). Interestingly, both values were obtained with the same strain, CMC 1981, suggesting that some peculiar problem could have occurred in its growth. However, the application of the other two algorithms for the ALPE estimation produced R^2^ values of 0.9956 in RPMI (Algorithm C) and 0.9501 in YEPD (Algorithm B). The case of this strain indicates that all three algorithms could be considered, using the one that best fits the growth behavior of the strain under investigation. This concept was further corroborated by considering that this strain shows the lowest average R^2^ values in both media. In general, Algorithm C performed better in RPMI (0.995 average R^2^), whereas Algorithm B was the best in YEPD with R^2^ of 0.98. As expected, the best performer also had the lowest standard deviations: 0.002 and 0.016 for Algorithm C in RPMI and Algorithm A in YEPD, respectively.

The application of the species-specific model was expected to be more challenging, due to the variability in cell size that affects the scattering of the light throughout the cell suspension. Furthermore, the variability of the cell growth style was considered another challenge to the three specific models. The specific models performed even better than the specific in RPMI, whereas in YEPD there were some lower R^2^ values, resulting in lower average R^2^ for the estimates with Algorithm A.

Even more challenging, the estimation of the initial cell number with the trans-specific model produced quite similar results in RPMI, where the average R^2^ of Algorithm A was 0.984 vs. 0.989 and 0.996 for the strain- and species-specific models, respectively. Algorithms B and C performed almost at the same level in all three models. In YEPD, the average R^2^ values were lower than in RPMI, but no major differences were detected in comparison with the estimates of the other two models. Namely, Algorithms A and B performed better than with the species-specific models and almost identically as the strain-specific model. Algorithm C was characterized by lower R^2^ values, ranging from 0.947 for the trans-specific to 0.954 for the species-specific model.

Finally, a cross validation was carried out with three strains (one from each species) that did not belong to the set of six strains used for the method set up. The trans-specific model and Algorithm A were employed to carry out the test in the most challenging conditions. Results show that the estimate of the initial cell density was still good with R^2^ values ranging from 0.9858 to 0.9952 (Figure 6c), indicating that the system works in a reliable way also with strains other than those employed to develop it. To make the use of these models swifter, we developed a MS Excel macro (provided as Supplementary Material File S3) that takes as input the data deriving from the plate reader and allows the automatic development of the regression curves and their usage.

## 4. Discussion

The aim of this study was to develop a new method able to estimate microbial cell density throughout a large range, using a common multi-plate reader equipped with a simple 405 nm filter, with a particular emphasis on the problem of determining low density inocula. The rationale of this work was that the cell density of the inoculum can be inferred from the kinetic parameters of the curve deriving from the growth of these cells. The results presented indicate that indeed the apparent lag phase, named ALPE, is inversely related to the initial cell number and therefore can be used for the density estimation from ca. 10^2^ to 10^7^ cells/mL^−1^. On the other hand, the optical density is a good estimator of cell density starting from values of 10^6^ cells/mL^−1^. These two findings were combined to produce an equation taking into account both ALPE and OD_405_ to produce an accurate estimate of the actual cell density. The fact that this approach was consistent with independent growths of the same strain used to calibrate the model was largely expected [27]. Less expected was the possibility to expand the use of this approach to the species level and then to different, although related, species [28,29]. The performance of the method is strongly dependent on the accurate determination of the ALPE, which in turn is strongly influenced by smooth transition of the lag and the exponential phase. For this reason, we developed three methods to estimate the ALPE, of which “Method A” has been proposed and discussed by several authors [26], whereas the two others have never been used so extensively. “Method C” performed well in many occasions and shares with “Method A” the advantage of not requiring any particular decision of the operator. On the contrary, “Method B” rests on the ability of the operator to determine a well working threshold. Although this method is definitely subjective, it performed well in many cases, indicating that it can be a good solution if the other two methods fail to find a consistent value for the ALPE. The ideal situation occurs when all, or at least two, ALPE estimates are concordant. For this reason, the control panel of the Excel Macro used for this study shows the simultaneous visualization of the three ALPE estimates to allow their comparison.

The proposed method was tested with cells simply deposited on the bottom of the plate well, simulating the situation of a biofilm, attached to a surface. This points out the possibility to use the method in both planktonic and sessile cell studies. Other methods have been proposed to estimate the cell numbers, such as real time PCR [8,10,30,31,32,33,34,35,36,37,38,39]. In comparison with this approach, the proposed method presents the advantage of lower costs and simpler technical setup because it has a faster learning curve. Other possible approaches to the determination of the cells adhering to a surface include their removal with glass beads [40] and sonication [41]. A limit of both these approaches is the possibility that the harsh conditions necessary to remove cells form the surface could damage or even kill a relevant part of them, leading to an obvious underestimate. On the other hand, it is possible that the proposed method will produce an overestimate if a large portion of dead cells are present in the well. However, this risk is only present when the cell density is well over 10^7^ cells mL^−1^, whereas the presence of dead cells does not affect the cell density estimate based on the ALPE, which relies only on the ability of viable cells to grow. From this point of view, the proposed method differs significantly from the other approaches of indirect total counts that do not differentiate between dead and living cells. Even the sophisticated approach with RT PCR is affected by this inability to discriminate between dead and live cells, which could be alleviated by using the much more complex and expensive approach of a real-time reverse-transcriptase (RT)-PCR [7,9,42,43,44,45,46].

One of the aims of this study was to produce a method amenable to being used in various settings, from research labs to the routine analyses in clinical, industrial and food industry laboratories. It could be used in online control of the microbial purity of industrial cultures, by applying this approach with media selecting against the fermentation starter, to assess the presence and quantity of contaminants. The advantage of this method is that its sensitivity would allow intervening in the culture very early after the contamination, thus giving the possibility to recover it, or at least to be aware of the contamination, before the commercialization of the fermentation products. Another potential application is the effective determination of the cell density in cases of septicemia, when the cell density is difficult to assess, or because it can be as low as a few hundred cells per mL [47,48]. Finally, for low density (<10^6^ cells mL^−1^) inoculations in the lab or at the pilot scale, the method can be applied with success since, for values around 10^3^ to 10^5^ cells mL^−1^, the proposed method is expected to work efficiently, as these values normally fall in the most central part of the calibration curve, typically characterized by few out-layers and good linearity.

## Figures and Tables

**Table 1:** Strains used in the study.

ID	Species	Source of Isolation
**CMC1968**	*C. albicans*	Pisa hospital
**CMC1959**	*C. albicans*	Udine hospital
**CMC2046**	*C. albicans*	Pisa hospital
**CMC1972**	*C. parapsilosis*	Pisa hospital
**CMC1981**	*C. parapsilosis*	Pisa hospital
**CMC1949**	*C. parapsilosis*	Udine hospital
**CMC1827**	*C. tropicalis*	Udine hospital
**CMC2052**	*C. tropicalis*	Pisa hospital
**CMC1839**	*C. tropicalis*	Udine hospital

**Table 2:** Correlation values of the cell densities in RPMI medium determined with Algorithms A–C and the three models (strain-specific, species-specific and trans-specific).

Model	Algorithm	CMC1968	CMC1959	CMC1972	CMC1981	CMC1827	CMC2052	ST.DEV
strain-specific	**A**	0.9985	0.9967	0.9995	0.9671	0.9781	0.9913	0.013
	**B**	0.9982	0.9832	0.9890	0.9561	0.9863	0.9929	0.015
	**C**	0.9963	0.9919	0.9936	0.9956	0.9956	0.9974	0.002
	**ST.DEV**	0.001	0.007	0.005	0.020	0.009	0.003	
species-specific	**A**	0.9983	0.9970	0.9915	0.9952	0.9967	0.9949	0.002
	**B**	0.9983	0.9833	0.9836	0.9509	0.9947	0.9989	0.018
	**C**	0.9801	0.9973	0.9949	0.9975	0.9995	0.9994	0.007
	**ST.DEV**	0.010	0.008	0.006	0.026	0.002	0.002	
Trans-specific	**A**	0.9974	0.9965	0.9808	0.9615	0.9834	0.9858	0.013
	**B**	0.9957	0.9833	0.9704	0.9567	0.9888	0.9894	0.015
	**C**	0.9967	0.9917	0.9907	0.9952	0.9969	0.9969	0.003
	**ST.DEV**	0.001	0.007	0.010	0.021	0.007	0.006	

**Table 3:** Correlation values of the cell densities in YPD medium determined with Algorithms A–C and the three models (strain-specific, species-specific and trans-specific).

Model	Algorithm	CMC1968	CMC1959	CMC1972	CMC1981	CMC1827	CMC2052	ST.DEV
strain-specific	**A**	0.9941	0.9899	0.9510	0.9476	0.9876	0.9789	0.020
	**B**	0.9908	0.9854	0.9738	0.9501	0.9897	0.9890	0.016
	**C**	0.9908	0.9917	0.9167	0.8168	0.9901	0.9941	0.072
	**ST.DEV**	0.002	0.003	0.029	0.076	0.001	0.008	
species-specific	**A**	0.9935	0.9908	0.9510	0.9439	0.9722	0.8305	0.061
	**B**	0.9892	0.9860	0.9728	0.9503	0.9985	0.9627	0.018
	**C**	0.9907	0.9885	0.9205	0.8328	0.9991	0.9940	0.066
	**ST.DEV**	0.002	0.002	0.026	0.066	0.015	0.087	
Trans-specific	**A**	0.9920	0.9895	0.9505	0.9488	0.9831	0.9775	0.019
	**B**	0.9858	0.9846	0.9744	0.9501	0.9891	0.9901	0.015
	**C**	0.9877	0.9862	0.9158	0.8160	0.9887	0.9858	0.070
	**ST.DEV**	0.003	0.003	0.029	0.077	0.003	0.006	

## Supplementary Materials

The following are available online at http://www.mdpi.com/2076-2607/7/2/32/s1, Figure S1. Optical Densities distribution at the inoculum time and after 24 h growth. (a,c,e) Inoculation time cell densities for the species: (a) *C. albicans* (CMC1959); (c) *C. parapsilosis* (CMC1972); and (e) *C. tropicalis* (CMC2052). (b,d,f) Optical density values after 24 h growth. RPMI medium is represented by the light-blue marker while YEPD by the orange one. Figure S2. g time values of the six strains: (a,b) g time of the two *C. albicans* strains CMC 1968 and CMC1959, respectively; (c,d) g time values of *C. parapsilosis* CMC1972 and CMC1981, respectively; and (e,f) g time of *C. tropicalis* CMC1827 and CMC2052, respectively. File S3. Macro used to analyze the dataset, developed with MS Excel.
