# Supplementary material for: High-Throughput Rapid and Inexpensive Assay for Quantitative Determination of Low Cell-Density Yeast Cultures
Source: Microorganisms. 2019 Jan 24;7(2):32. doi: 10.3390/microorganisms7020032 (PMC6406537; doi:10.3390/microorganisms7020032)
Supplement: Supplementary file 1 [file microorganisms-07-00032-s001.zip › Supplementary Material S3 Description.pdf]

NB: The MS Excel macro developed to analyse the data is available upon request, free of charge.

MACRO_DESCRIPTION

Paste your data in ‘Raw Dada’ excel sheet (columns A to M) and start ‘Matrix_Generator’ macro by clicking the corresponding button. If data were previously analysed, first clear the content on the right by pressing the “Clear” button.
‘Matrix_Generator’ macro will organize the data, as generated by “Magellan”, in columns. Each column created corresponds to a succession of OD values (belonging to the same well) collected at subsequent timepoints.
In the example below is shown the A1-well data sorting to form the corresponding column of 4 subsequent timepoints (i.e.: 5 minutes).

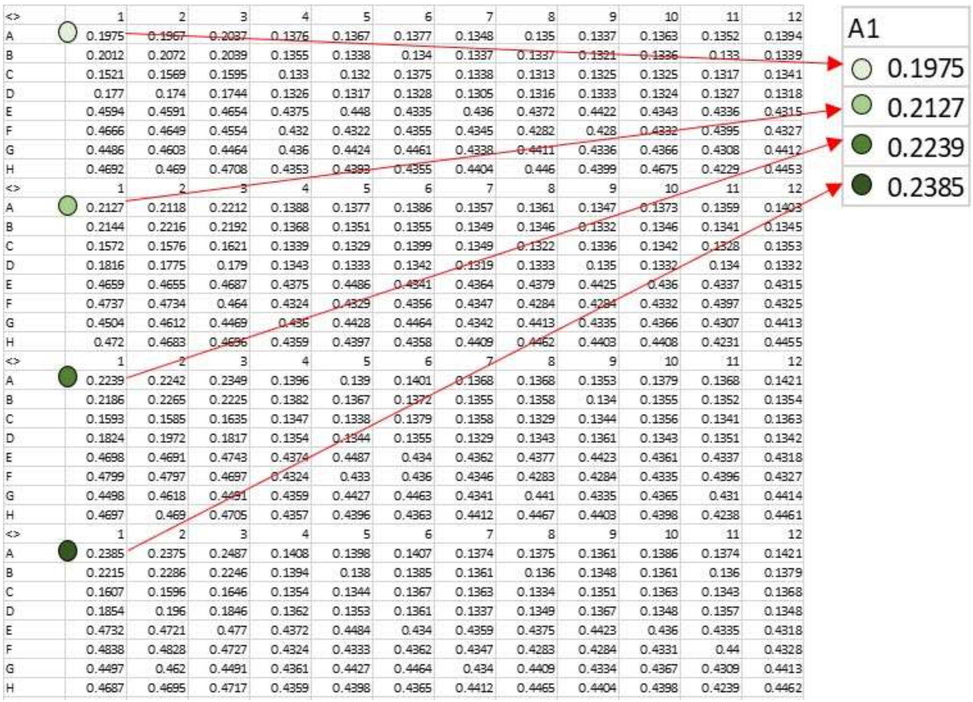

<>	1	2	3	4	5	6	7	8	9	10	11	12
A	0.1975	0.1967	0.2037	0.1376	0.1367	0.1377	0.1348	0.135	0.1337	0.1363	0.1352	0.1394
B	0.2012	0.2072	0.2039	0.1355	0.1338	0.134	0.1337	0.1337	0.1321	0.1336	0.133	0.1339
C	0.1521	0.1569	0.1595	0.133	0.132	0.1375	0.1338	0.1313	0.1325	0.1325	0.1317	0.1341
D	0.177	0.174	0.1744	0.1326	0.1317	0.1328	0.1305	0.1316	0.1333	0.1324	0.1327	0.1318
E	0.4594	0.4591	0.4654	0.4375	0.448	0.4335	0.436	0.4372	0.4422	0.4343	0.4336	0.4315
F	0.4666	0.4649	0.4554	0.432	0.4322	0.4355	0.4345	0.4282	0.428	0.4332	0.4395	0.4327
G	0.4486	0.4603	0.4464	0.436	0.4424	0.4461	0.4338	0.4411	0.4336	0.4366	0.4308	0.4412
H	0.4692	0.469	0.4708	0.4353	0.4393	0.4355	0.4404	0.446	0.4399	0.4675	0.4229	0.4453
<>	1	2	3	4	5	6	7	8	9	10	11	12
A	0.2127	0.2118	0.2212	0.1388	0.1377	0.1386	0.1357	0.1361	0.1347	0.1373	0.1359	0.1403
B	0.2144	0.2216	0.2192	0.1368	0.1351	0.1355	0.1349	0.1346	0.1332	0.1346	0.1341	0.1345
C	0.1572	0.1576	0.1621	0.1339	0.1329	0.1399	0.1349	0.1322	0.1336	0.1342	0.1328	0.1353
D	0.1816	0.1775	0.179	0.1343	0.1333	0.1342	0.1319	0.1333	0.135	0.1332	0.134	0.1332
E	0.4659	0.4655	0.4687	0.4375	0.4486	0.4341	0.4364	0.4379	0.4425	0.436	0.4337	0.4315
F	0.4737	0.4734	0.464	0.4324	0.4329	0.4356	0.4347	0.4284	0.4284	0.4332	0.4397	0.4325
G	0.4504	0.4612	0.4469	0.436	0.4428	0.4464	0.4342	0.4413	0.4335	0.4366	0.4307	0.4413
H	0.472	0.4683	0.4696	0.4359	0.4397	0.4358	0.4409	0.4462	0.4403	0.4408	0.4231	0.4455
<>	1	2	3	4	5	6	7	8	9	10	11	12
A	0.2239	0.2242	0.2349	0.1396	0.139	0.1401	0.1368	0.1368	0.1353	0.1379	0.1368	0.1421
B	0.2186	0.2265	0.2225	0.1382	0.1367	0.1372	0.1355	0.1358	0.134	0.1355	0.1352	0.1354
C	0.1593	0.1585	0.1635	0.1347	0.1338	0.1379	0.1358	0.1329	0.1344	0.1356	0.1341	0.1363
D	0.1824	0.1972	0.1817	0.1354	0.1344	0.1355	0.1329	0.1343	0.1361	0.1343	0.1351	0.1342
E	0.4698	0.4691	0.4743	0.4374	0.4487	0.434	0.4362	0.4377	0.4423	0.4361	0.4337	0.4318
F	0.4799	0.4797	0.4697	0.4324	0.433	0.436	0.4346	0.4283	0.4284	0.4335	0.4396	0.4327
G	0.4498	0.4618	0.4491	0.4359	0.4427	0.4463	0.4341	0.441	0.4335	0.4365	0.431	0.4414
H	0.4697	0.469	0.4705	0.4357	0.4396	0.4363	0.4412	0.4467	0.4403	0.4398	0.4238	0.4461
<>	1	2	3	4	5	6	7	8	9	10	11	12
A	0.2385	0.2375	0.2487	0.1408	0.1398	0.1407	0.1374	0.1375	0.1361	0.1386	0.1374	0.1421
B	0.2215	0.2286	0.2246	0.1394	0.138	0.1385	0.1361	0.136	0.1348	0.1361	0.136	0.1379
C	0.1607	0.1596	0.1646	0.1354	0.1344	0.1367	0.1363	0.1334	0.1351	0.1363	0.1343	0.1368
D	0.1854	0.196	0.1846	0.1362	0.1353	0.1361	0.1337	0.1349	0.1367	0.1348	0.1357	0.1348
E	0.4732	0.4721	0.477	0.4372	0.4484	0.434	0.4359	0.4375	0.4423	0.436	0.4335	0.4318
F	0.4838	0.4828	0.4727	0.4324	0.4333	0.4362	0.4347	0.4283	0.4284	0.4331	0.44	0.4328
G	0.4497	0.462	0.4491	0.4361	0.4427	0.4464	0.434	0.4409	0.4334	0.4367	0.4309	0.4413
H	0.4687	0.4695	0.4717	0.4359	0.4398	0.4365	0.4412	0.4465	0.4404	0.4398	0.4239	0.4462

A1
0.1975
0.2127
0.2239
0.2385

In order to transfer the data to ‘Data Analysis’ excel sheet, click on “Data Transfer” button (1) in the same sheet and the data will appear starting from column E.
The macro “Alpe_Def” will be started by clicking on “ALPE Estimation” button (2).

Parameters setting

-ALGORITHM A:
set on “YES” (3) for the automatic search of starting and ending point of the exponential phase. If the selection is set on “NO” (3), determine manually starting (Start EXP) and ending point (End EXP).
delta t (4): corresponds to the number of points to consider for searching the exponential phase. To be set the analyst.

threshold R^2 (5): the R^2 of the tangent to the exponential phase. To be modulate by the analyst based on data observation.
threshold slope (6): sets the minimum tangent's slope. To be modulate by the analyst based on data observation.
-ALGORITHM B: set **threshold [%]** (7) considering the end of the Lag Phase as the time at which the initial optical density exceeds a predetermined growth.

-ALGORITHM C: set **moving window width** (8) sets the width of the sliding window necessary to calculate the incremental rates. To be modulate by the analyst based on data observation.

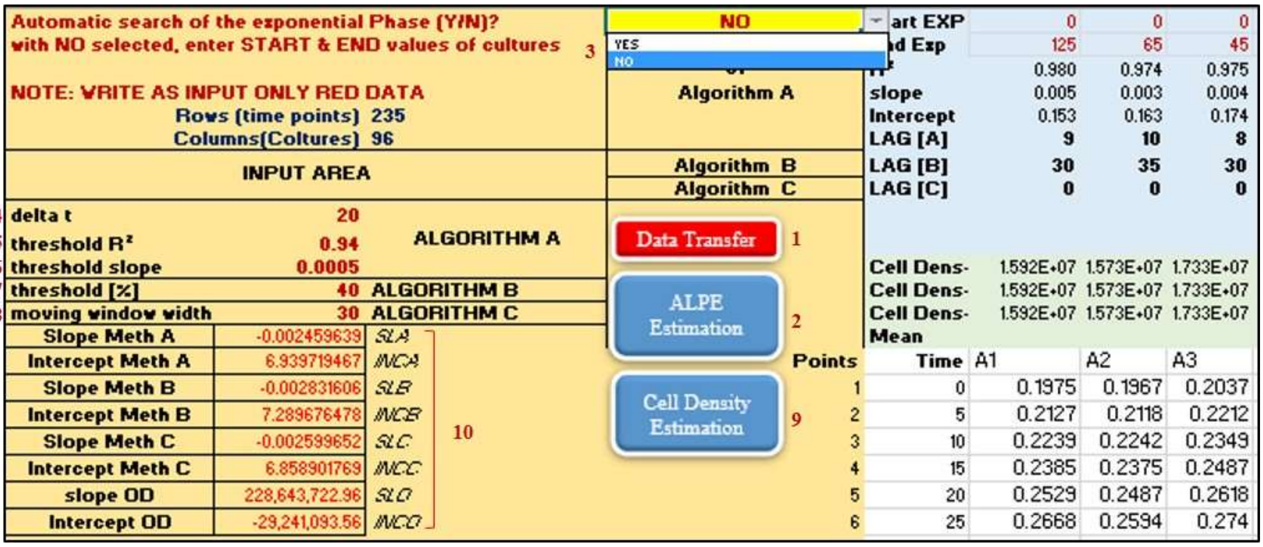

Once ALPE length is calculated, is possible to determine the cell density by clicking on "Cell Density Estimation" button (9). Table (10) presents the parameters of the model for the cell density calculation.
We built 3 different *Candida* Species-specific models (each one was developed using 2 strains of the same species) that could be pasted where necessary in the appropriate table (10).

-*Candida albicans*:

Slope Meth A	-0.004569479
Intercept Meth A	6.711482924
Slope Meth B	-0.005014698
Intercept Meth B	7.014083306
Slope Meth C	-0.003820833
Intercept Meth C	6.495291554
slope OD	149,472,632.03
Intercept OD	-20,324,959.77

-*Candida parapsilosis*:

**Slope Meth A**	-0.002459639
**Intercept Meth A**	6.939719467
**Slope Meth B**	-0.002831606
**Intercept Meth B**	7.289676478
**Slope Meth C**	-0.002599652
**Intercept Meth C**	6.858901769
**slope OD**	228,643,722.96
**Intercept OD**	-29,241,093.56

-*Candida tropicalis*:

**Slope Meth A**	-0.005819253
**Intercept Meth A**	7.211986867
**Slope Meth B**	-0.00542361
**Intercept Meth B**	7.667376687
**Slope Meth C**	-0.00495723
**Intercept Meth C**	6.545923652
**slope OD**	113,380,755.56
**Intercept OD**	-14,070,394.06
