# Supplementary figures and images for: High-Throughput Rapid and Inexpensive Assay for Quantitative Determination of Low Cell-Density Yeast Cultures
Source: Microorganisms. 2019 Jan 24;7(2):32. doi: 10.3390/microorganisms7020032 (PMC6406537; doi:10.3390/microorganisms7020032)

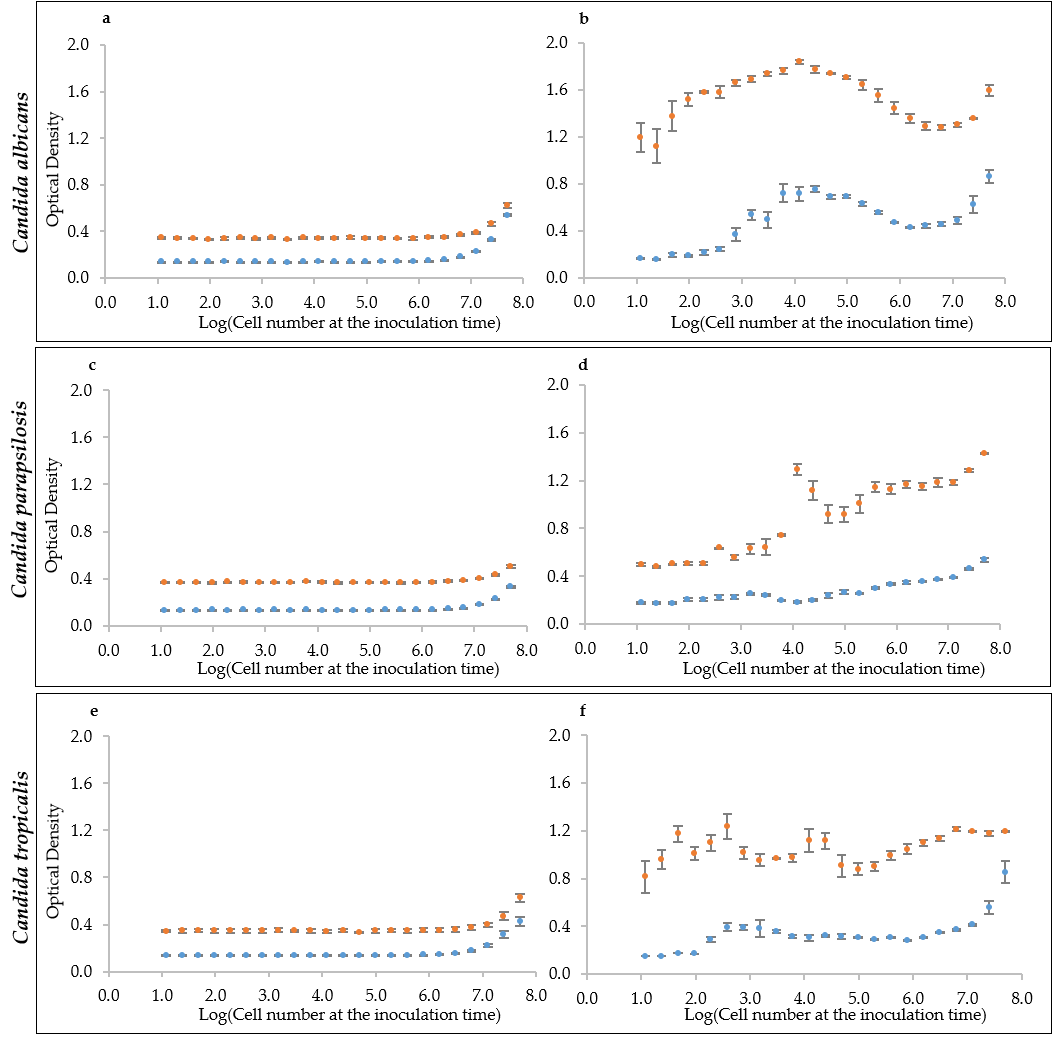

Supplement: Supplementary file 1 [file microorganisms-07-00032-s001.zip › Figure S1.tif]

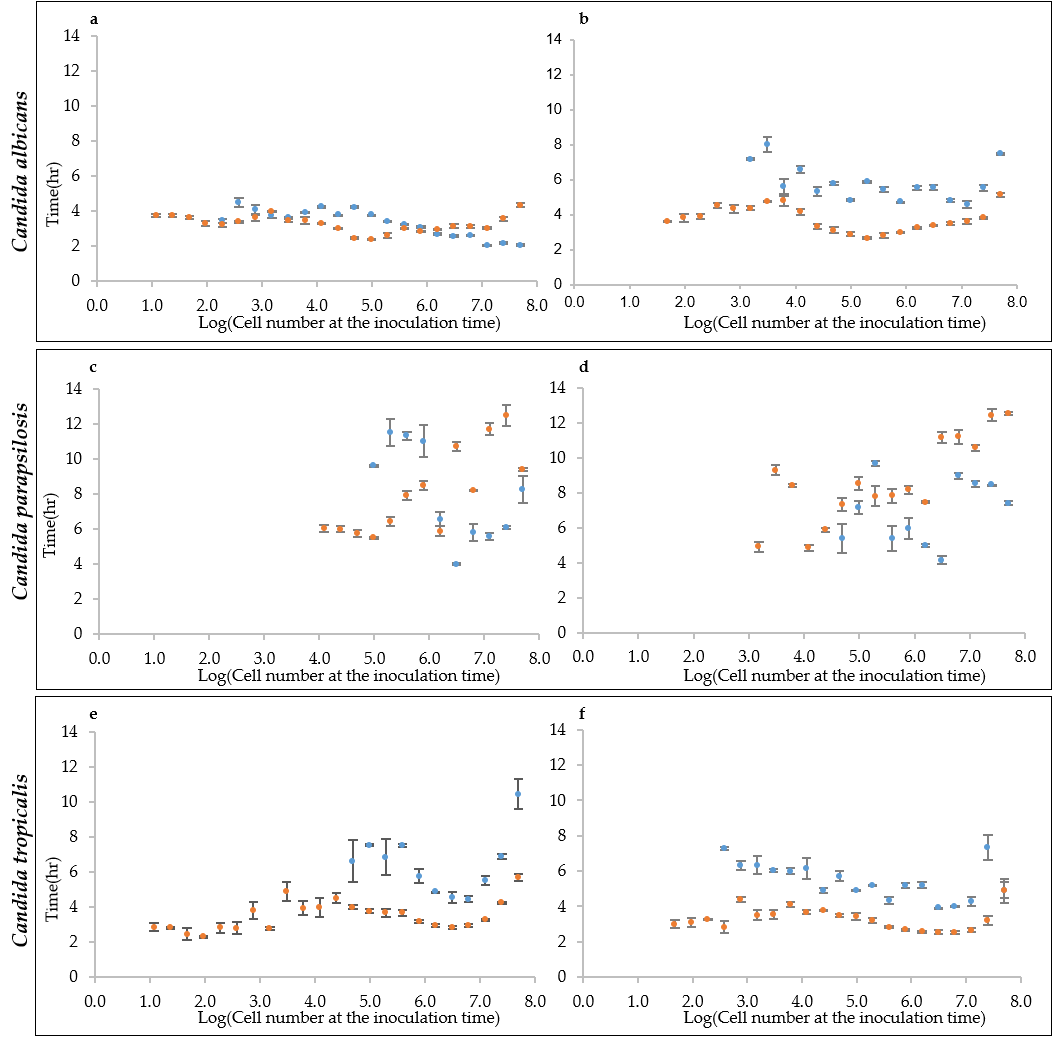

Supplement: Supplementary file 1 [file microorganisms-07-00032-s001.zip › Figure S2.tif]
